# Temperature‐Controlled Radiofrequency Treatment of the Nasal Valve in Patients With Nasal Obstruction: Long‐Term Outcomes

**DOI:** 10.1002/ohn.1118

**Published:** 2025-01-17

**Authors:** Joseph K. Han, Jon N. Rosenthal, Chad M. McDuffie, David M. Yen, Nadim B. Bikhazi, Venkata Vasu Kakarlapudi, Stacey L. Silvers

**Affiliations:** ^1^ Department of Otolaryngology–Head and Neck Surgery Eastern Virginia Medical School Norfolk Virginia USA; ^2^ ENT and Allergy Associates of Florida Coral Springs Florida USA; ^3^ ENT Associates of Texas McKinney Texas USA; ^4^ Specialty Physician Associates Bethlehem Pennsylvania USA; ^5^ Ogden Clinic Ogden Utah USA; ^6^ Advanced ENT and Allergy New Albany Indiana USA; ^7^ Madison ENT & Facial Plastic Surgery New York New York USA

**Keywords:** nasal airway obstruction, nasal valve dysfunction, sleep obstruction, temperature‐controlled radiofrequency

## Abstract

**Objective:**

To evaluate the efficacy, safety, and durability of temperature‐controlled radiofrequency (TCRF) treatment of the nasal valve in patients with severe or extreme nasal airway obstruction (NAO).

**Study Design:**

A long‐term, prospective, multicenter, single‐blind, randomized controlled trial.

**Setting:**

Sixteen otolaryngologic clinics and academic centers.

**Methods:**

Patients received TCRF treatment on the lateral nasal valve. All patients were followed through 3 years. Outcome measures included the Nasal Obstruction Symptom Evaluation (NOSE) Scale, Epworth Sleepiness Scale (ESS), and adverse events (AEs). Treatment responders were defined as a ≥1 reduction in severity class or ≥20% reduction in NOSE score.

**Results:**

Out of 108 patients who received TCRF treatment, 54 reached the 3‐year follow‐up timepoint. The baseline mean NOSE score was 76.3 (95% confidence interval [CI], 73.6 to 79.1). The 3‐year NOSE score treatment effect was −49.4 ([95% CI, −56.5 to −42.4]; *P* < .001) a 64.7% improvement from baseline; 88.7% of patients were responders. Most patients reported significant improvements in sleep post‐treatment with a mean ESS score of 4.5 (95% CI, 3.4 to 5.7) at 3 years compared to 10.3 (95% CI, 9.2 to 11.4) at baseline.

**Conclusion:**

Treatment with the TCRF device for nasal valve obstruction resulted in sustained improvements in nasal obstruction symptoms and sleep quality over a 3‐year period without any serious AEs. These findings support the long‐term benefits and sustained improvements in symptoms in patients with NAO.

Nasal airway obstruction (NAO) is a prevalent issue that significantly impacts patients’ quality of life and is frequently associated with bothersome symptoms such as congestion, headaches, and poor sleep quality.^1^, ^2^ NAO remains a clinical challenge to treat, often requiring multiple treatment modalities and persistent medication usage. Contributing factors such as turbinate hypertrophy, septal deviation, nasal polyps, rhinitis, and other structures all play a role in NAO.^3^, ^4^ However, one of the main causes of NAO is nasal valve dysfunction (NVD),^5^ characterized by the narrowing or collapse of the nasal valve (NV), leading to difficulty in breathing through the nose. NVD can be further classified into static NVD or dynamic NVD. Static NVD typically includes a high septal deviation or an enlarged turbinate, whereas dynamic NVD includes a weakness in the integrity of the upper lateral cartilage and nasal side wall as well as an abnormality in the upper lateral cartilage and lateral nasal wall upon inspiration.^6^

To alleviate the symptoms of NAO with NVD, the main goal of the clinician should be to improve airflow through the nasal passage while preserving the integrity of the NV. This can be accomplished through various methods. Nonsurgical methods include nasal dilators, nasal sprays, allergy medications, or breath right strips; however, these require continuous usage and frequent replacement. Surgical approaches like septorhinoplasty with/without turbinate reduction, cartilage grafting, or implants are beneficial, but may not be appropriate for some patients and carry surgical risks and increased procedural costs.^4^, ^7^

Treatment of NAO and NVD conditions using minimally invasive approaches (i.e., suspension sutures, implants, or stents) may be a middle‐ground approach as it permits the patient to get a structural change with fewer risks compared to surgical approaches, while also reducing their continued reliance on medications. It may also be of preference for patients due to the lower financial burden, avoidance of anesthesia, faster recovery time, and fewer workdays lost compared with surgery.^8^, ^9^

Temperature‐controlled radiofrequency (TCRF) treatment is a minimally invasive procedure that can be performed in an office or ambulatory surgery center and can be used as a first‐line treatment, or as an alternative to revision surgery for patients with persistent NAO after a previous rhinoplasty or NV repair.^10^, ^11^ TCRF treatment provides controlled energy delivery to the submucosal layer of the lateral nasal wall which induces tissue remodeling through the contraction of collagen proteins, stimulating the production of new collagen proteins, resulting in an increased diameter of the nasal passage, allowing for better airflow.^12^

Clinical studies conducted to date have shown TCRF to have a durable treatment effect. In a pivotal study, the TCRF treatment effect was shown to be significant for up to 4 years.^13^ Two single‐arm studies also demonstrated a significant decrease from baseline in Nasal Obstruction Symptom Evaluation (NOSE) scores at 12 months and 2 years.^14^, ^15^ A recent meta‐analysis including 451 patients also found a significant improvement in NOSE scores among patients treated with radiofrequency treatment compared to pretreatment and control groups.^16^ Previously reported results from this present randomized controlled trial (RCT) demonstrated that TRCF treatment of the internal NV had significant improvements in NAO symptoms observed at 3 months that were sustained through 24 months.^17^, ^18^, ^19^

Here, we report the final results of this long‐term extension study in which patients were followed through 3 years to evaluate the duration of the treatment effect of TCRF treatment of the lateral NV in patients with NAO. Long‐term durability studies can aid in policy decision‐making regarding the utilization of TCRF in patient management.

## Methods and Materials

### Study Design and Patient Population

This was a multicenter, prospective, single‐blinded, RCT that evaluated the effectiveness and safety of the TCRF treatment of the NV in adult patients with severe or extreme NAO. The primary study included 118 patients from 16 otolaryngologic clinics and academic centers in the United States. This study was conducted in accordance with the Declaration of Helsinki, in compliance with Good Clinical Practice and local regulations, and approved by the WCG Institutional Review Board (IRB) and Eastern Virginia Medical School IRB. The clinical trial was registered at ClinicalTrials.gov (NCT04549545). All patients enrolled provided written informed consent.

Participants were randomized to either active treatment or a sham procedure, with the study maintaining blinding for patients to minimize bias. After a designated follow‐up period, patients in the sham group were eligible for crossover to the active treatment, ensuring all had the opportunity to receive the therapeutic intervention. Further details on randomization and crossover procedures are available in previous publications.^17^, ^18^, ^19^

Eligible subjects ≥22 years who met all inclusion/exclusion criteria (as previously described),^17^, ^18^, ^19^ were enrolled between August 2020 and December 2020 and followed up in the primary study through 2 years. Those completing the 2‐year study were invited to participate in the extended follow‐up study to collect outcomes through 3 years.

Treatment was performed by board‐certified otolaryngologists. After administration of local anesthesia, TCRF treatment of the NV was applied bilaterally to the nasal mucosa at the junction of the upper and lower lateral cartilage of the lateral nasal wall using the VivAer® ARC stylus (Aerin Medical), with up to 4 nonoverlapping applications and no retreatment allowed.

### Study Objective and Assessments

This extension study evaluated the long‐term durability of efficacy and safety of the TCRF procedure for patients with severe/extreme NAO (based on pretreatment NOSE scores) due to NVD through 3 years post‐treatment. Efficacy endpoints included the percentage of patients who were treatment responders, defined as a ≥20% reduction (i.e., improvement) in total NOSE score or ≥1 NOSE severity category improvement compared to baseline; change from baseline in total NOSE score; change from baseline in each NOSE score category (nasal congestion, nasal blockage, trouble breathing, trouble sleeping, and getting enough air during exercise); change from baseline in total Epworth Sleepiness Scale (ESS) score; the proportion of participants reporting a change in use of medication for nasal obstruction. Demographic information was collected for each patient, including age, sex, race, and ethnicity. Medical and nasal histories were collected at baseline, and nasal assessments were conducted during each study visit. Patients were excluded from the study if they had any additional ENT procedures post‐treatment.

A NOSE score was completed at each follow‐up visit. The NOSE score, a validated patient‐reported outcome measure, was used to measure the outcomes of patients treated for NAO.^20^ Symptom categories in the questionnaire include nasal congestion, nasal blockage, trouble breathing, trouble sleeping, and less air during exercise. Each category was scored using a 5‐point Likert scale to make a total score range of 0 through 100. The severity of symptoms was classified as mild (range, 5‐25), moderate (range, 30‐50), severe (range, 55‐75), or extreme (range, 80‐100).

The ESS score is a self‐reported questionnaire that assesses the severity of daytime sleepiness in patients.^21^ The survey consists of 8 questions using a 4‐point scale (0‐3) to assess the likelihood of dozing or falling asleep while engaging in 8 different activities with daytime sleep propensity rated as no chance (0), slight (1), moderate (2), or high chance (3). The total sum of the scores to classify respondents having lower normal daytime sleepiness (range 0‐5), higher normal daytime sleepiness (range, 6‐10), mild excessive daytime sleepiness (range, 11‐12), moderate excessive daytime sleepiness (range, 13‐15), and severe excessive daytime sleepiness (range, 16‐24).

This was a pragmatic study and medication use was not dictated. Current use of medication, nasal devices, or other therapies for symptoms of nasal obstruction, including medication name, frequency, and dose were captured at baseline and each follow‐up visit. Medications were classified into separate categories (antihistamines, decongestants, leukotriene inhibitors, steroid nasal sprays, anticholinergic nasal sprays, immunotherapy, and other). The proportion of participants reporting an increase, decrease, or no change in the use of medications was summarized.

Adverse events (AEs), including type, incidence, duration, severity, and relatedness to the procedure and/or device were collected at each time point, starting from screening through 36 months. Patients with AEs were followed for 30 days or until the event was resolved.

### Statistical Methods

A sample size of approximately 120 patients was planned for the initial study. Analyses performed after the initial unblinding and evaluation of the primary endpoint were conducted using the combined active treatment group consisting of all participants who received active TCRF treatment (ie, the index active treatment arm and the sham crossover active treatment).

Statistical analyses were performed using SAS version 9.4 (SAS Institute Inc). Outcome measures were summarized using frequencies and percentages for categorical measures and mean, adjusted (least squares) mean, median, standard deviation, minimum, and maximum for continuous variables. Ninety‐five percent confidence intervals (CIs) for mean differences between treatment groups were included.

Other analyses include descriptive summaries of patient scores on the NOSE Scale, ESS, and the change in the frequency of medication use. Descriptive summaries were assessed at 3, 6, 12, 24, and 36 months post‐procedure.

An additional subanalysis was conducted on patients with NVD (dynamic, static, complex, or mixed), as defined previously.^18^ Patients who discontinued the study early or were lost‐to‐follow‐up were assessed (Early Exit Analysis) based on their NOSE or ESS score at their last study visit.

## Results

### Patient Characteristics

**Figure 1:**
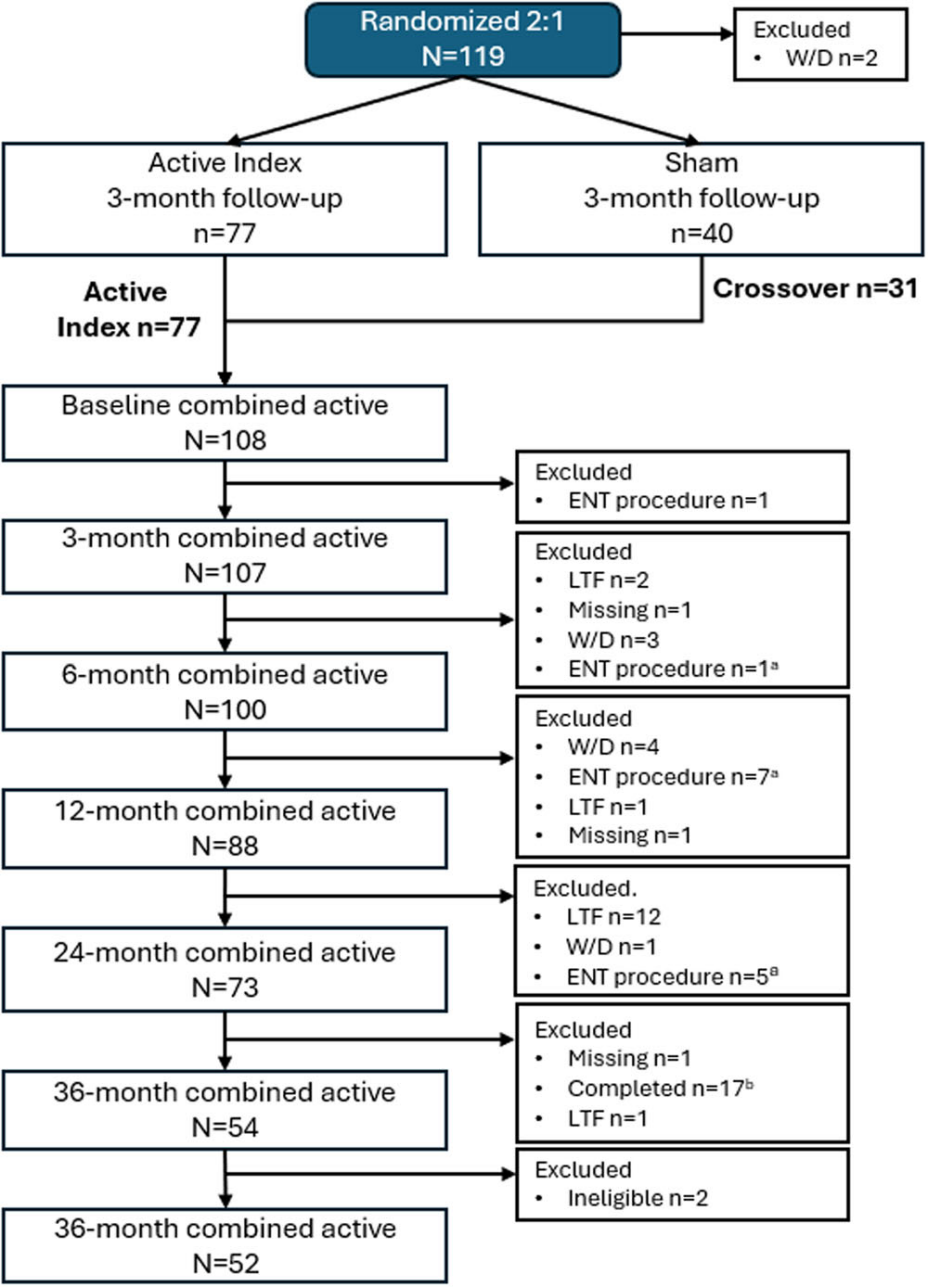
CONSORT flowchart of patient disposition. ^a^Patient had additional ENT procedures. ^b^Completed 2 year study. CONSORT, Consolidated Standards of Reporting Trials; LTF, lost to follow‐up; W/D, withdrawn.

In the initial study, a total of 118 patients were enrolled, and 108 patients received active TCRF treatment (77 from the index active arm and 31 from the crossover arm) and 106 patients were included in the primary analysis (2 patients from the crossover arm in the initial study were excluded from the analysis due to pretreatment NOSE scores < 55). Of the 73 patients in the combined active treatment group in the initial study, 55 agreed to participate in the 3‐year extended follow‐up study, 54 were included in the intent‐to‐treat population, and 52 in the per‐protocol population (Figure 1). Demographic and baseline characteristics are summarized in Table 1. Nearly 20% of subjects reported having obstructive sleep apnea (OSA) at baseline. As reported previously, 14 patients were excluded from the study due to additional ENT procedures, most were completed within the first year (Supplemental Table S1, available online).^17^ Nine patients had additional procedures that targeted the inferior turbinate. Only 4 patients were documented as having additional procedures related to the NV, supporting the notion that TCRF is effective at treating NAO with NVD.

**Table 1:** Demographics and Baseline Characteristics (Intent‐to‐treat)

Characteristic	Combined active 3 mo cohort	Combined active 36 mo cohort
No.	108	54
Female	66 (61.11%)	34 (62.96%)
Age, mean (SD), y	48.55 (12.28)	49.24 (11.85)
BMI, mean (SD), kg/m^2^	28.99 (5.87)	30.13 (5.92)
Race, No. (%)		
American Indian or Alaska Native	2 (1.85)	2 (3.70)
Asian	2 (1.85)	1 (1.85)
Black or African American	6 (5.56)	3 (5.56)
White	96 (88.89)	48 (88.89)
Declined choices	2 (1.85)	0 (0.0)
Medical history, No. (%)		
Nasal surgery^a^	32 (29.63)	15 (27.78)
Allergic rhinitis^b^	43 (39.81)	26 (48.15)
Nonallergic rhinitis^b^	15 (13.89)	9 (16.67)
Sinus disease^c^	15 (13.89)	4 (7.41)
Obstructive sleep apnea	21 (19.44)	10 (18.52)
NOSE score, mean (SD)^d^	76.34 (14.32)	76.48 (12.31)
Nasal valve collapse mechanism, No. (%)		
Bilateral dynamic	51 (47.2)	29 (50.7)
Bilateral static	34 (31.5)	14 (26.0)
Mixed	15 (13.9)	5 (15.1)
Complex^e^	8 (7.4)	4 (8.2)

Abbreviations: BMI, body mass index; NOSE, nasal obstruction symptom evaluation; SD, standard deviation.

^a^ Includes inferior/middle turbinate reduction/excision, septoplasty, rhinoplasty, sinuplasty, and functional endoscopic sinus surgery.

^b^ Based on patient or provider knowledge, no tests were performed as part of the trial.

^c^ A combination of acute sinusitis or chronic rhinosinusitis.

^d^ Includes active index and crossover arm.

^e^ Complex includes patients with a different or mixed mechanism on each side, that is, dynamic on one side, static on the other; or static and dynamic on one side, static or dynamic on the other side.

### Efficacy Outcomes

**Figure 2:**
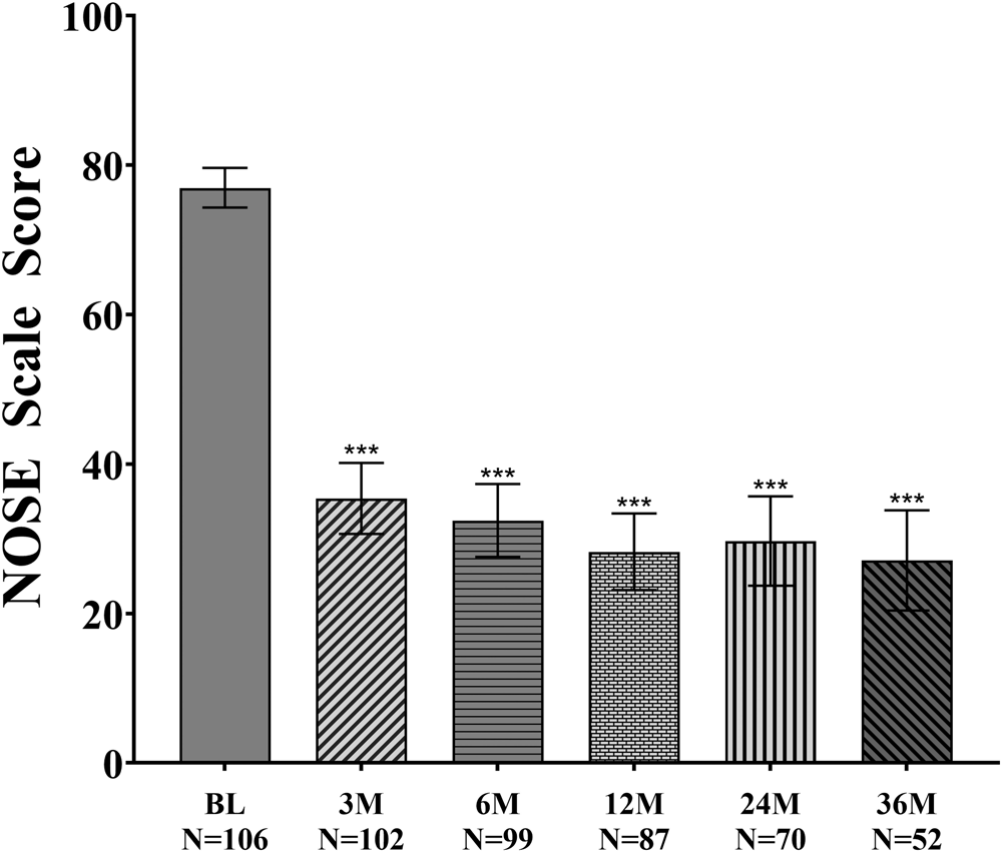
Adjusted mean change in NOSE score at BL, 3M, 6M, 12M, 24M, and 36M. ***P < .001 versus BL. Bars represent 95% CI. BL, baseline; CI, confidence interval; M, months; NOSE, Nasal Obstruction Symptom Evaluation.

**Figure 3:**
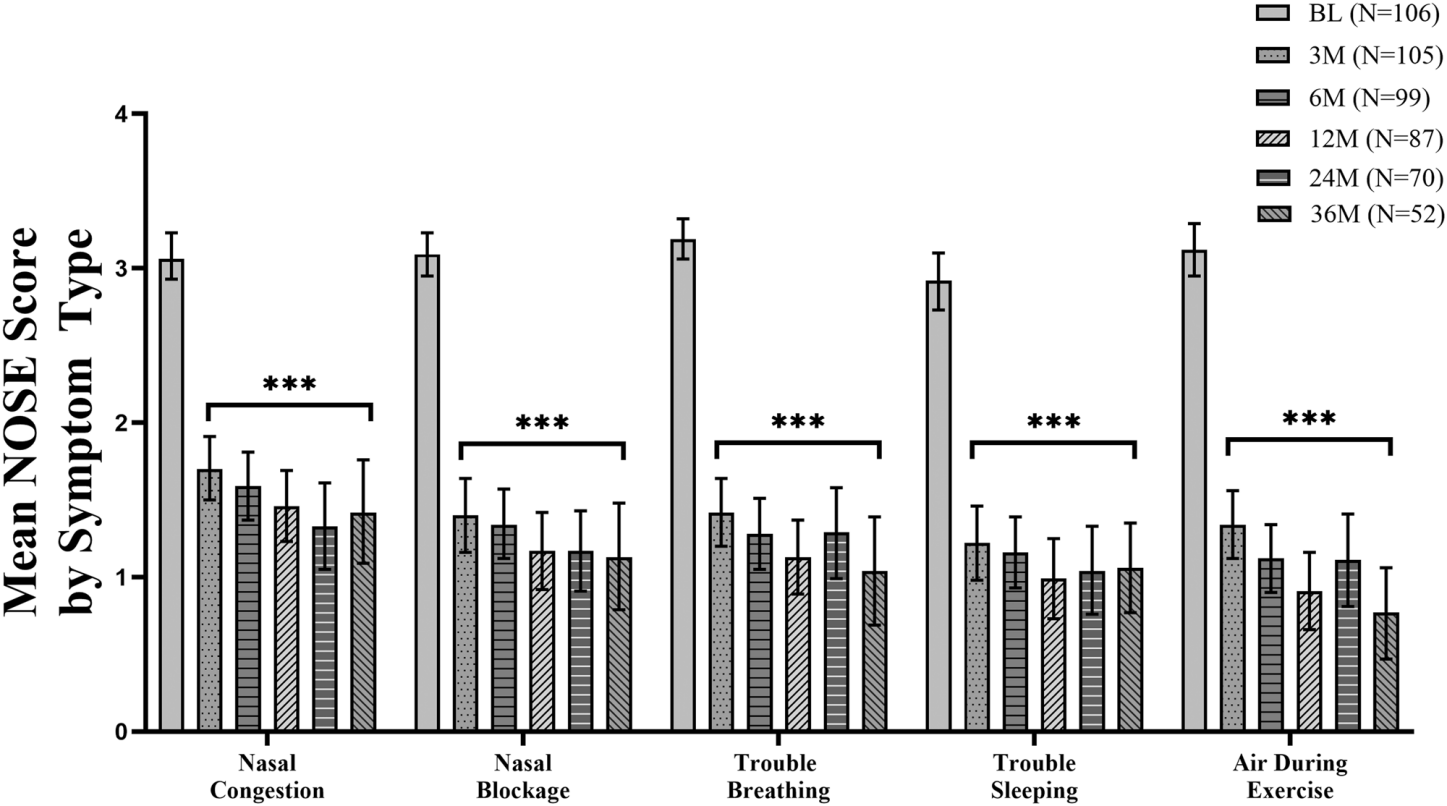
Participants' mean score for each NOSE score component at baseline through 36M following TCRF treatment. ***P < .001 versus BL. Bars represent 95% CI. BL, baseline; CI, confidence interval; M, months; NOSE, Nasal Obstruction Symptom Evaluation; TCRF, temperature‐controlled radiofrequency.

**Figure 4:**
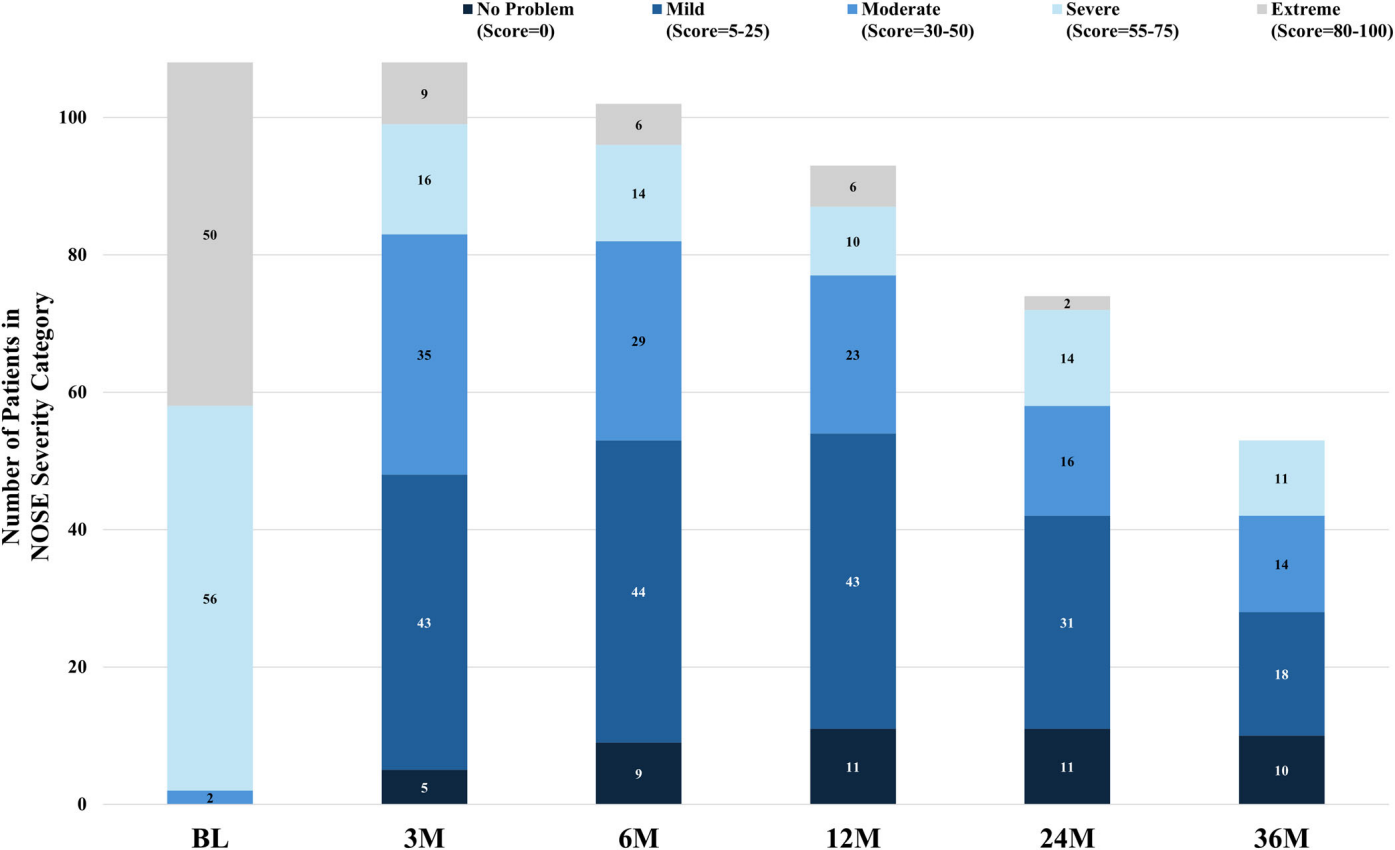
Number of participants at each NOSE severity category at baseline through 36M following TCRF treatment. BL, baseline; M, months; NOSE, Nasal Obstruction Symptom Evaluation; TCRF, temperature‐controlled radiofrequency.

TCRF treatment continued to have a durable effect on improving NAO symptoms through 3 years, with a responder rate of 87%, consistent with earlier findings.^17^ The NOSE score improved from baseline at all follow‐up timepoints and was sustained through 3 years: an adjusted mean NOSE score of 27.1 (95% CI, 20.4‐33.8) compared to baseline of 76.9 (95% CI, 74.4 to 79.6); mean difference, ‐49.5 (95% CI, −56.6 to −42.4; *P* < .001) (Figure 2). These data represent a 64.6% improvement in the NOSE score from baseline at 3 years. Significant improvements were demonstrated in all 5 subcomponents of the NOSE score (nasal congestion, nasal blockage, trouble breathing, trouble sleeping, and getting enough air during exercise) at each follow‐up timepoint through 3 years compared to baseline; *P* < .001 (Figure 3). At baseline, all patients had either severe or extreme NAO symptoms improving to 53.8% having none or only mild NAO symptoms and 48.7% having severe or extreme NAO symptoms at 3 years (Figure 4).

**Figure 5:**
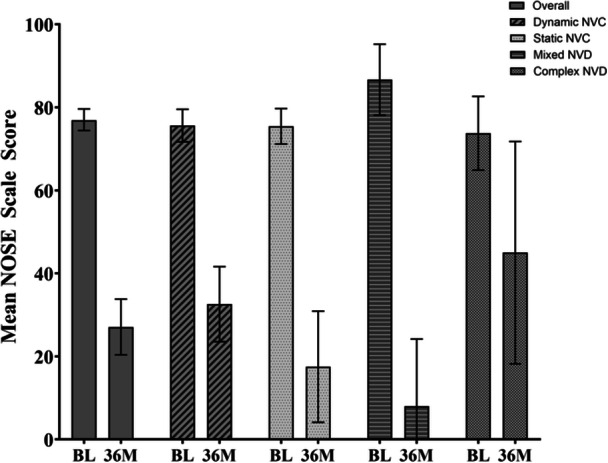
Adjusted mean NOSE score based on type of NVD. Bars represent the 95% CI. BL, baseline; CI, confidence interval; M, months; NOSE, Nasal Obstruction Symptom Evaluation; NVD, nasal valve dysfunction.

To determine if NVD played a role in the efficacy of TCRF treatment, a subgroup analysis was performed according to the mechanism of valve collapse. Patients were divided into 4 groups (bilateral dynamic, bilateral static, mixed, and complex). The mechanism of NV collapse did not appear to affect NOSE Scale score outcomes in any of the 4 groups which were comparable at all follow‐up time points through 3 years (Figure 5). Consistent with earlier findings, the adjusted mean change in NOSE Scale score at 3 years was −43.8 (95% CI, −53.85 to −33.73) for bilateral dynamic NVD, −56.4 (95% CI, ‐67.94 to ‐44.91) for static NVD, −77 (95% CI, −91.29 to −62.71) for mixed NVD, and −32.5 (95% CI, −73.8 to 8.84) for complex NVD.

**Figure 6:**
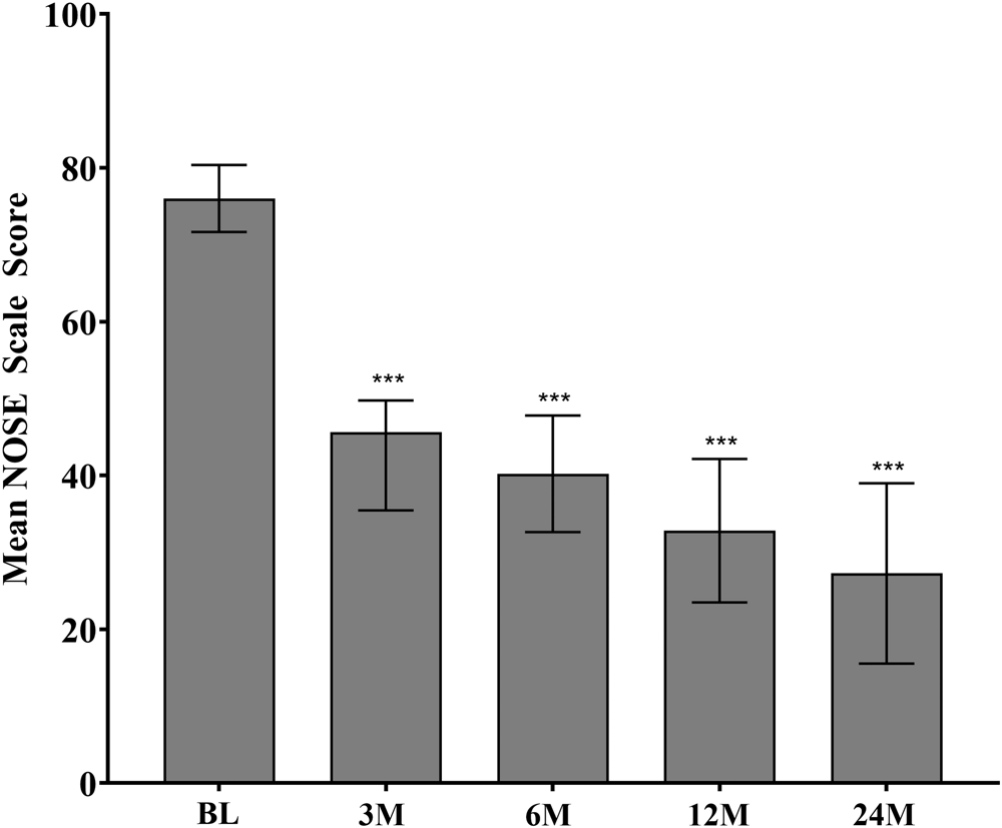
Adjusted mean NOSE score for early exit cohort. ***P < .001 versus BL. Bars represent 95% CI. BL, baseline; CI, confidence interval; M, months; NOSE, Nasal Obstruction Symptom Evaluation.

An additional subanalysis of patients (N = 56) who exited the study prior to the 36‐month visit was conducted to determine if the high attrition rate had any impact on the efficacy outcomes. Of the subjects who exited the study, the mean NOSE Scale score was 76.0 (95% CI, 71.64 to 80.36) at baseline compared with 27.3 (95% CI, 15.53 to 38.97) at their last study visit with a mean difference of ‐49.6 (95% CI, −63.21 to −36.29) (Figure 6).

**Figure 7:**
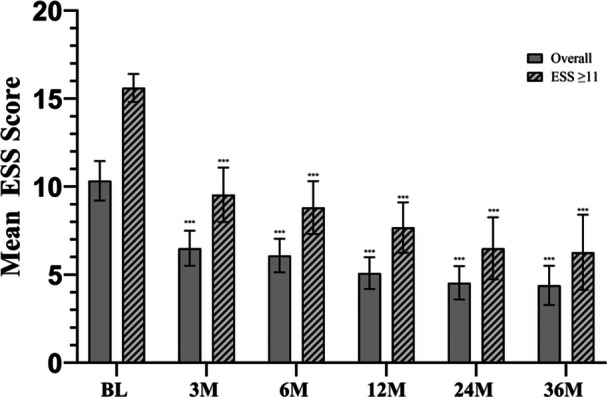
Adjusted mean ESS scores overall and for subpopulations with ≥11 ESS score (mild‐to‐severe excessive daytime sleepiness) at baseline through 36M. ***P < 0.001 versus BL. Bars represent the 95% CI. BL, baseline; CI, confidence interval; ESS, Epworth Sleepiness Scale; M, months.

The impact of TCRF treatment on daytime sleepiness showed that TCRF significantly improved mean ESS scores at 3 years compared to baseline (4.5 vs. 10.3; difference, −4.85 (95% CI, −6.26 to −3.45); *P* < .001 at all timepoints (Figure 7). In the 51 (48.1%) patients with baseline ESS scores of ≥11 or higher, the improvement was greater; the mean score at baseline was 15.6 (95% CI, 14.8 to 16.4) compared with 6.27, indicating higher normal range daytime sleepiness (95% CI, 4.14 to 8.41) at 3 years, with an adjusted mean change in score at 3 years of −8.8 (95% CI, −11.0 to −6.6). Similarly, the mean ESS scores were significantly improved in patients (N = 55) who exited the study prior to the 36‐month follow‐up visit (Supplemental Figure S1, available online).

### Medication Usage

At year 3, patients continued to have sustained relief of NAO symptoms, as demonstrated by their decrease in medication use. For most participants (91.8%) medication usage remained the same or had been reduced or discontinued at 3 years (Table 2). When analyzed by type of medication, 53.6% of patients decreased or stopped oral antihistamines, 69.2% decreased or stopped decongestants, 42.9% decreased or stopped leukotriene inhibitors, and 68% decreased or stopped intranasal steroid sprays. Two subjects (4%) overall reported an increase in medication use; 1 subject had an increase in oral antihistamine medicine, and 1 subject had an increase in intranasal steroid spray. Likewise, the majority of patients who exited the study prior to the 36‐month follow‐up visit did not increase their medication or device usage as of their last study visit (Supplemental Table S2, available online).

**Table 2:** Change in Nasal Medication and NAO Device Usage at Baseline and 3 Years

Medication class	Used at baseline (N = 52), N (%)	Medication change category	No. of patients N (%)^a^	Medication started after baseline N (%)^b^
Antihistamines	28 (53.8)			3 (2.8)
		No change in medication	12 (42.9)	
		Increased dose	1 (3.6)	
		Decreased dose	5 (17.9)	
		Stopped medication	10 (35.7)	
Decongestants	13 (24.5)			2 (1.9)
		No change in medication	4 (30.8)	
		Increased dose	0 (0)	
		Decreased dose	0 (0)	
		Stopped medication	9 (69.2)	
Leukotriene inhibitors	7 (13.2)			1 (0.9)
		No change in medication	4 (57.1)	
		Increased dose	0 (0)	
		Decreased dose	1 (14.3)	
		Stopped medication	2 (28.6)	
Nasal steroid sprays^c^	24 (46.2)			0 (0)
		No change in medication	7 (28)	
		Increased dose	1 (4)	
		Decreased dose	2 (8)	
		Stopped medication	14 (58.3)	
Nasal anticholinergic sprays	2 (3.8)			0 (0)
		No change in medication	0 (0)	
		Increased dose	0 (0)	
		Decreased dose	1 (50)	
		Stopped medication	1 (50)	
Immunotherapy	2 (3.8)			0 (0.0)
		No change in medication	1 (50)	
		Increased dose	0 (0)	
		Decreased dose	1 (50)	
		Stopped medication	0 (0)	
Other^d^	2 (3.8)			1 (0.9)
		No change in medication	1 (50)	
		Increased dose	0 (0)	
		Decreased dose	0 (0)	
		Stopped medication	1 (50)	

Abbreviation: NAO, nasal airway obstruction.

^a^ Percentage was calculated by using the number of patients who reported using each medication at baseline as the denominator.

^b^ Percentage was calculated by using the total number of patients (N = 52) as the denominator.

^c^ Steroid sprays include intranasal compound sprays.

^d^ Other equals the sum of “antihistamine/decongestant,” expectorant, and other combination medications.

### Safety

All device/procedure‐related AEs have been previously reported.^17^ No additional procedure‐ or device‐related AEs were reported in the interval after the 6‐month through 3 years. No serious procedure‐ or device‐related AEs were reported at any time during the study.

## Discussion

Treating patients with severe nasal obstruction continues to remain a clinical challenge. NAO can have a significant impact on patients, causing a multitude of burdens. Patients with NAO often experience a diminished quality of life due to symptoms such as nasal congestion, difficulty breathing through the nose, snoring, disrupted sleep, or increased daytime sleepiness. The role of NAO has also been implicated as a contributing factor to sleep‐disordered breathing and OSA.^22^, ^23^

NAO is typically managed through medication and/or nasal devices as first‐line treatment. The goal of medical management is primarily to decrease mucosal inflammation and edema using pharmacological (topical and/or systemic) treatments and/or mechanically open the nasal passage.^24^, ^25^ However, once all other interventions are exhausted, patients with persistent NAO symptoms will often seek additional ENT procedures to find relief, with surgery often being the last resort. The AAO‐HNS notes that office‐based techniques, including radiofrequency treatment, can stabilize the NV and optimize NV collapse patient outcomes.^26^ The AAO‐HNS also notes that nasal surgery may benefit patients either as a standalone treatment,^27^, ^28^ or by improving compliance with oral appliances and continuous positive airway pressure (CPAP) devices, by reducing nasal airway resistance.^29^, ^30^, ^31^ TCRF treatment may bridge the gap between nasal surgery and OSA, by improving nasal airway resistance, NAO symptoms, and sleep quality through a less invasive method compared to surgery.

Results from this study demonstrate that the treatment effect of TCRF is efficacious and sustained through 3 years. The responder rate and NOSE Scale scores at 3 years were significantly improved compared to baseline and consistent with reports from other studies.^13^, ^14^ The NOSE scale score of patients with dynamic, static, mixed, or complex NVD as a contributing factor was also assessed and found to have no impact on treatment effect.

Patients with sleep‐disordered breathing or OSA often experience excessive daytime sleepiness, which can negatively affect cognition, mood, daily functioning, and overall well‐being.^32^ Nearly half (48%) of patients in this study had ESS scores of ≥11 at baseline. Daytime sleepiness remained significantly improved at 3 years, as demonstrated by lower ESS scores. In addition, the NOSE subscore for sleep was significantly improved compared to baseline indicating that TCRF treatment of NVD may potentially offer an additional benefit to improve sleep. Many patients also continued to have less reliance on medication, even 3‐years post‐procedure.

To account for the potential bias in patients who exited the study or were lost‐to‐follow‐up, we assessed the difference in mean NOSE scores, ESS scores, and medication/device usage between the participants who reached the 3‐year follow‐up and those who exited the study compared with their last follow‐up visit and found no notable differences. Most patients who exited the study had clinically meaningful improvements in their NAO symptoms and ESS scores, and only a small subset of patients went on to have additional ENT procedures. Therefore, it is unlikely that their decision to not participate in the 3‐year extension study was due to the lack of symptomatic improvement.

In addition to the safety and efficacy, the use of TCRF as a minimally invasive alternative to surgery may offer potential benefits related to decreased costs and fewer risks compared to surgery and as the procedure can be performed in an office setting, may enable avoidance of general anesthesia and prolonged recovery time. A recently published budget impact model estimated that patients with NAO with NVD treated with TCRF could save an average of $3,531 over 4 years due to lower procedure costs and complication rates than surgical intervention.^33^ Simmons et al published a retrospective chart review of TCRF treatment in 37 patients and found an average of 55.6% improvement in their NOSE scores (or −34.7 change from baseline).^10^ Kang et al reported similar results in their systematic review and meta‐analysis, finding an average change from baseline of −44.5 (95% CI, −49.2 to −39.8) at 3 months, and −56.4 (95% CI, −62.4 to −50.3) at 2 years.^16^

The results of this study support the overall safety of TCRF use for NVD. No serious AEs occurred in the study in both the short and long‐term follow‐up time points, likely due to the controlled energy delivery which is mucosal sparing.

While the results of this study are favorable, the limitations should be acknowledged. First, patients were not restricted in their nasal medication usage as an enrollment criterion throughout the study. Second, the study was limited to the treatment of the NV alone and may not represent a real‐world scenario in which multiple contributors to NAO may be treated with TCRF. Further studies looking at the potential additive value of TCRF treatment of septal swell body and inferior turbinate hypertrophy in addition to the NV may better demonstrate the full potential effect. Additionally, clinically objective measures for nasal airflow, such as rhinomanometry, acoustic rhinometry, and peak nasal inspiratory flow, were not studied and should be considered for future studies to assess the quantitative impact of TCRF. Finally, we acknowledge that we did not capture CPAP data between the initial procedure and final follow‐up, which may be a confounding factor to the changes demonstrated in ESS and would provide useful information regarding the benefit of TCRF to OSA patients.

## Conclusion

TCRF treatment is an effective, safe method with long‐term durability for improving NAO in patients with NVD and should be considered as a potential alternative to surgical approaches to NV repair when evaluating treatment options with patients as part of a shared decision‐making process.

## Author Contributions

**Joseph K. Han**, contributed to the design, conduct, analysis, drafted, reviewed, and approved the final manuscript; **Jon N. Rosenthal**, contributed to the conduct, analysis, and critical review and approval of the manuscript; **Chad M. McDuffie**, contributed to the conduct, analysis, and critical review and approval of the manuscript; **David M. Yen**, contributed to the conduct, analysis, and critical review and approval of the manuscript; **Nadim B. Bikhazi**, contributed to the conduct, analysis, and critical review and approval of the manuscript; **Venkata Vasu Kakarlapudi**, contributed to the conduct, analysis, and critical review and approval of the manuscript; **Stacey L. Silvers**, contributed to the design, conduct, analysis, drafted, reviewed, and approved the final manuscript. All authors had access to the study data and have reviewed and approved the final manuscript.

## Disclosures

### Competing interests

Joseph K. Han: Consultant for Aerin Medical, Medtronic, Intersect ENT, Genentech, Sanofi Genzyme, Astra Zeneca, and GlaxoSmithKline. Jon N. Rosenthal: None. Chad M. McDuffie: None. David M. Yen: Received research funding from Aerin Medical and is a consultant and/or has received research funding for/from: 3‐D Matrix, Astra Zeneca, AventaMed, Cyrano Therapeutics, Eli Lilly, Evidera, GlaxoSmithKline, Lyra Therapeutics, Medtronic, Neubio North America, Neurent Medical, OptiNose, Pocket Naloxone, Regeneron, Sanofi Genzyme, Sound Health, Spirair, Tympanogen; and has stock options in Cyrano Therapeutics, Diag‐Nose Medical, and Sound Health. Nadim B. Bikhazi: None. Venkata Vasu Kakarlapudi: None. Stacey L. Silvers: Consultant for Aerin Medical, 3D matrix, and Lyra Therapeutics and on the medical advisory board for STS stent.

### Funding source

This study was funded by Aerin Medical. The sponsor also provided funding for data analysis and manuscript preparation assistance; however, the final content was solely the responsibility of the authors, and the authors did not receive direct payments for the authorship and/or publication of this article.

## Supporting information

Supporting information.
