# Supplementary material for: Temperature‐Controlled Radiofrequency Treatment of the Nasal Valve in Patients With Nasal Obstruction: Long‐Term Outcomes
Source: Otolaryngol Head Neck Surg. 2025 Jan 17;172(4):1214–23. doi: 10.1002/ohn.1118 (PMC11947858; doi:10.1002/ohn.1118)
Supplement: Supplementary file 1 — Supporting information. [file OHN-172-1214-s001.docx]

**Supplemental Table 1.** Details for patients with additional nasal procedures

|  |  |  |  |  |  | **NOSE Scale Score** | | |
| --- | --- | --- | --- | --- | --- | --- | --- | --- |
| **Pt** | **Nasal valve collapse at BL** | **Septal Deviation at BL** | **Reason for Procedure** | **Additional procedure after TCRF treatment** | **Days after TCRF treatment** | **BL^a^** | **Last Score Prior to Additional**  **procedure** | **Responder prior to Additional**  **procedure^b^** |
| 1 | Static | No | **Septal deviation, nasal congestion, and NAO​** | **Septorhinoplasty** | 266 | 100 | 80 | Yes |
| 2 | Dynamic | Yes | **Persistent nasal obstruction​** | **Septoplasty, turbinate reduction** | 56 | 75 | 90 | No |
| 3 | Mixed | No | **chronic pansinusitis** | **Septoplasty,**  **turbinate reduction, FESS** | 164 | 80 | 45 | yes |
| 4 | Dynamic | No | **Persistent nasal congestion, turbinate hypertrophy** | **Repeat VivAer, Turbinate Reduction** | 203 | 85 | 65 | yes |
| 5 | Static | Yes | **non allergic rhinosinusitis, deviated septum** | **Balloon sinuplasty, septoplasty** | 226 | 70 | 50 | yes |
| 6 | Mixed | No | **turbinate hypertrophy, moderate NVC** | **Septoplasty, turbinate reduction, unspecified “sinus surgery”** | 252 | 90 | 75 | No |
| 7 | Dynamic | Yes | **septal deviation, turbinate hypertrophy** | **turbinate reduction** | 199 | 90 | 50 | Yes |
| 8 | Dynamic | No | **Turbinate hypertrophy, septal deviation, narrowing MM** | **Bilateral NV surgery, turbinate reduction** | 250 | 85 | 70 | No |
| 9 | Static | No | **chronic sinus disease** | **Balloon sinuplasty** | 234 | 70 | 60 | No |
| 10 | Dynamic | Yes | **turbinate hypertrophy** | **turbinate reduction** | 477 | 55 | 55 | No |
| 11 | Mixed | No | **NAO, drainage, pressure, Chronic rhinitis** | **Latera implant, RhinAer** | 613 | 85 | 15 | Yes |
| 12 | Dynamic | No | **chronic sinusitis, elective FESS** | **Septoplasty, FESS** | 678 | 65 | 35 | Yes |
| 13 | Dynamic | No | **chronic sinusitis, turbinate hypertrophy** | **Septoplasty, turbinate reduction** | 695 | 90 | 35 | Yes |
| 14 | Mixed | Yes | **Persistent Nasal Obstruction, Sleep Disordered Breathing, Uvula Elongated, Turbinate Hypertrophy** | **Turbinate reduction, uvulectomy** | 510 | 100 | 65 | Yes |

Abbreviations: BL = baseline; FESS = functional endoscopic sinus surgery; ITH = inferior turbinate hypertrophy; NAO = nasal airway obstruction; NV = nasal valve; NVC = nasal valve collapse; TCRF = temperature-controlled radiofrequency.

^a^ Baseline was at study start for Active Index arm and Crossover Baseline for Sham Control

^b^ Responder per the study definition prior to the additional procedure.

**Supplemental Table 2**. Change in Medication Use for Nasal Obstruction Symptoms at Last Study Visit

| **Medication Category** | **Medication Use** | **Statistic** | **Early Exit Cohort (N=56)** |
| --- | --- | --- | --- |
| Oral antihistamines | Decreased | n (%) | 2 (3.70%) |
|  | Stayed the same | n (%) | 14 (25.93%) |
|  | Increased | n (%) | 1 (1.85%) |
|  | Not Applicable | n (%) | 37 (68.52%) |
| Oral decongestants | Decreased | n (%) | 2 (3.70%) |
|  | Stayed the same | n (%) | 2 (3.70%) |
|  | Not Applicable | n (%) | 50 (92.59%) |
| Oral compound medications (antihistamine/decongestant) | Decreased | n (%) | 1 (1.85%) |
|  | Stayed the same | n (%) | 1 (1.85%) |
|  | Not Applicable | n (%) | 52 (96.30%) |
| Oral leukotriene inhibitors | Decreased | n (%) | 1 (1.85%) |
|  | Stayed the same | n (%) | 4 (7.41%) |
|  | Not Applicable | n (%) | 49 (90.74%) |
| Intra-nasal antihistamine sprays | Decreased | n (%) | 1 (1.85%) |
|  | Not Applicable | n (%) | 53 (98.15%) |
| Intra-nasal decongestant sprays | Decreased | n (%) | 4 (7.41%) |
|  | Stayed the same | n (%) | 2 (3.70%) |
|  | Increased | n (%) | 1 (1.85%) |
|  | Not Applicable | n (%) | 47 (87.04%) |
| Intra-nasal steroid sprays | Decreased | n (%) | 5 (9.26%) |
|  | Stayed the same | n (%) | 9 (16.67%) |
|  | Increased | n (%) | 1 (1.85%) |
|  | Not Applicable | n (%) | 39 (72.22%) |
| Intra-nasal anticholinergic sprays | Decreased | n (%) | 2 (3.70%) |
|  | Increased | n (%) | 1 (1.85%) |
|  | Not Applicable | n (%) | 51 (94.44%) |
| Intra-nasal mast cell stabilizer sprays | Decreased | n (%) | 1 (1.85%) |
|  | Not Applicable | n (%) | 53 (98.15%) |
| Breathe Right Strips | Decreased | n (%) | 7 (12.96%) |
|  | Stayed the same | n (%) | 1 (1.85%) |
|  | Not Applicable | n (%) | 46 (85.19%) |
| Insert nasal cones | Decreased | n (%) | 2 (3.70%) |
|  | Not Applicable | n (%) | 52 (96.30%) |
| Saline | Decreased | n (%) | 6 (11.11%) |
|  | Stayed the same | n (%) | 14 (25.93%) |
|  | Increased | n (%) | 2 (3.70%) |
|  | Not Applicable | n (%) | 32 (59.26%) |
| Other | Decreased | n (%) | 3 (5.56%) |
|  | Stayed the same | n (%) | 1 (1.85%) |
|  | Increased | n (%) | 1 (1.85%) |
|  | Not Applicable | n (%) | 49 (90.74%) |

**Supplemental Figure 1.** Adjusted mean Epworth Sleepiness Scale (ESS) scores for the early exit analysis subgroup at baseline through 36 months. **p-value <0.05 vs Baseline. Bars represent the 95% CI.
